# Analysis of economic forecasting in the post-epidemic era: evidence from China

**DOI:** 10.1038/s41598-022-19011-z

**Published:** 2023-02-15

**Authors:** Xin Li

**Affiliations:** School of Mathematics and Statistics, Beijing Technology and Business University, Beijing, 100048 China

**Keywords:** Applied mathematics, Statistics

## Abstract

This paper presents a predictive analysis of the Chinese economy in the post-epidemic era. Five major public health emergencies historically similar to the COVID-19 epidemic are used as the control group, and a fuzzy mathematical model is applied to forecast and analyze China’s economy after the COVID-19 epidemic. The forecast results show that China’s overall economy will have recovered to the pre-epidemic level in about 1 year, with the fastest recovery in individual economic indicators, followed by government final consumption and imports, then CPI, fiscal revenue, exports and money supply, and the slowest recovery in employment. Finally, a combination of all the parties makes policies and recommendations for China’s economic and social development in the post-epidemic era.

## Introduction

The COVID-19 epidemic spread dramatically throughout 2020 and became a global pandemic. By April 2022, 95 million people had been infected worldwide, with 6 million deaths attributable to the epidemic. To date the virus continues to mutate and spread. Bontempi et al.^1^ found that the increase in cases was proportional to the total number of imports and exports in Italy, leading to the conclusion that international trade was the main cause of the epidemic over and above other factors such as population, economy, environment and pollution transport. China is a major export and import country, and coupled with the long incubation period and high level of infectiousness of the virus, the outbreak has proved the most serious public health emergency since the founding of China. Under the strong leadership of the Communist Party Central Committee, China has achieved significant results in coordinating epidemic prevention and control alongside maintaining economic and social development over the last couple of years. However, the arrival of new variants of the coronavirus looks set to become the norm, and so undoubtedly the greatest challenge facing China and other countries in the post-epidemic era is how to ensure long-term economic and social prosperity when living alongside the new variants.

Throughout history, epidemics have been known to slow down economic growth or even cause a major impact on the economy. Therefore, it is of great significance to study economic recovery following the disaster. He^2^ used economic forecasting methods to build a mathematical model of SARS. He used the 0.618 method, the time series index smoothing technique, and the Wendt method to process the data longitudinally and horizontally, and derived the economic recovery of China following the SARS epidemic. Tang and Wu^3–5^ studied post-disaster economic recovery theory from a macro perspective, and provided an analytical study of the overall economic recovery following the Tangshan and Wenchuan earthquake. Huang^6^ used fuzzy mathematics to construct an economic forecasting model to predict the economic recovery after the 2011 Tohoku earthquake and tsunami in Japan. Ruan^7^ used the consumer confidence index (CCI) as a starting point to construct a model to analyze the prior prognostic effect of the CCI of COVID-19 epidemic on many aspects of the macroeconomy. Webb^8^ used a questionnaire to allow people to subjectively compare changes in customer numbers and profitability after the disaster to predict business recovery in a county in Florida after the earthquake. Ewing^9^ studied employment growth in Texas after the earthquake. Lam^10^ investigated the recovery rate of industries in New Orleans after Hurricane Katrina, with early recovery proven in the science and technology sector but late recovery in the wholesale and retail sector. Xiao^11^ used the Marx Index to study the impact of flooding on employment and agriculture, and simulated the change in values to obtain economic recovery by finding a city very similar to the affected city as a control group. These scholars, on the other hand, analyze and study the post-disaster recovery of a specific industry or field to derive the economic recovery of a particular industry or field. Bin^12^ concluded that the economy will recover rather than fall into a long-term recession by building a joint model to predict economic recovery for countries, such as the United States and India. Dayang Jiang^13^ used an ARIMA model to predict the GDP of the Chinese economy without an epidemic based on the theory of elasticity economy and related forecasting methods, verifying that strong economic resilience plays an important role in a country's economic response to a major shock. It is evident that recovery forecasting of the post-disaster economy has become a hot research topic among scholars.

The recovery of a post-disaster economy also has a lot to do with epidemic prevention and control policies, financial policies to stimulate the economy or other external factors. The Global Risk Report 2021 released by the “World Economic Forum” points out that the “livelihood crisis” caused by the coronavirus epidemic is the main risk facing the world for the next 2 years. The “livelihood crisis” refers to the increase in the cost of living due to the continuous increase in the number of employee shortagese, and also the restructuring of industries, leading to changes in the world landscape. In addition, non-pharmaceutical public health interventions such as trade imports and exports, and reduced cross-border movement of people, which are the most important drivers of the spread of the epidemic, as well as people’s anxieties and some governmental foot bans, will also affect the economic recovery. For example, Usadhi^14^ found that the government and social resources play a large role in post-disaster economic recovery. If the government develops a more rational recovery system, it will be more beneficial to economic and social recovery. Keogh-Brown^15^ analyzed the GDP, import and export of countries affected by the SARS epidemic. Tourism and retail sales were also analyzed and the impact of the epidemic was found to only last a few months rather than a few years. Sylvain^16^ studies the worldwide economic recovery from the 2008 financial crisis over the last 20 years and concluded that only half of the sample countries had an economic recovery that was consistent with a V–U–L type of economic recovery graph. Rebret^17^ examines whether non-pharmacological public health interventions during the 1918 Spanish Flu pandemic prolonged mortality, and found that school closures, bans on gatherings, and non-pharmacological interventions by means of quarantine prolonged deaths but did not eliminate them. Austan^18^ examines the drivers of economic slowdown due to the epidemic, and found that legal quarantine restrictions accounted for only a small part of the problem, with other factors such as people’s anxieties and a shift in consumer buying habits. Kayode^19^ studies the relationship between epidemiological data and socioeconomic determinants by modeling the transmission dynamics of the epidemic in different countries and their different approaches to suppressing the epidemic. Qiu^20^ uses an empirical model to study the impact of socioeconomic factors on COVID-19, using China as an example transmission and concluded that some determinants have rich implications for the current efforts to contain the epidemic. The scholars' study reveals that government intervention, public confidence in the market, and international trade transactions can affect the recovery, but not all of them prolong the recovery time, and if the policies and guidance are reasonably formulated, the economy can be equally quick to recover. In addition, good and appropriate financial policies and support policies can also lead to rapid economic recovery. Wunhong^21^ reveals that the epidemic had a huge impact on small and medium-sized enterprises (SMEs) and provides short- and long-term policy recommendations to help SMEs following the epidemic. Li^22^ concludes that the Chinese government successfully assessed public risk and launched a clear and timely vaccination policy. Yawen^23^ explores how to strike a balance between epidemic prevention and control and sustainable economic development, and proposes that China dynamically adjusts the intensity of transportation restrictions and economic stimulus policies according to regional differences.

Synthesizing the contents and conclusions of previous studies, this paper argues that as China was the main driver behind world economic growth following the financial crisis in 2008, it is necessary to study the recovery of China’s economy in the post-epidemic era. Most existing articles related to the economic recovery in the post-epidemic era have only conducted modeling studies concerning one or several factors, or they have focused on the recovery of several industries without a more comprehensive economic forecasting model. This paper differs in that we use the method of fuzzy mathematics^24,25^ and include more factors in the same model to provide a systematic forecasting analysis. Fuzzy mathematics was proposed in “Fuzzy Set Theory” published by Prof. Zadeh in 1965, and has developed various analytical methods such as fuzzy decision making, fuzzy prediction and fuzzy control, which have been effectively applied in many fields such as medicine, economic management and control theory. Based on the concept of fuzzy sets, this paper selects five major historical public health emergencies that are similar to the COVID-19 epidemic, and uses the data related to these five events to predict the combined economic as well as individual economic variables of the COVID-19 epidemic. Since all five events differ from the current outbreak to varying degrees, there is no clear criterion for classifying the differences between outbreaks, so the problem of weighting coefficients between the five selected outbreaks and the COVID-19 outbreak can be more accurately predicted using a fuzzy mathematical model based on Euclidean geometry. At the same time, in an attempt to include all the factors affecting economic recovery, we determine eight macroeconomic indicators and six epidemic characteristic indicators that can cover more subjective and objective factors. Finally, we verify the accuracy of the prediction model based on our empirical results, and thereafter propose policies suggestions suitable for China's economic development in the post-epidemic era. The most innovative point of this paper is the establishment of a prediction model for the degree of economic recovery from the COVID-19 epidemic through analysis of similar epidemic data in history, which enables a systematic prediction of the recovery time of integrated and individual economic indicators.

## Data selection and modeling principle

### Data selection

With so many factors interplaying in the post-epidemic era, a systematic research approach is needed to forecast economic recovery. In terms of comprehensive economic forecasting, this paper attempts to use major international public health emergencies similar to the COVID-19 epidemic to make forecasts, and how to determine the weights of each event is key to producing more accurate forecasting results. To date the most popular weighting methods used in former research are the mean square error inverse weighting method, entropy weighting method and optimization method, but they are limited by the sample size and too much calculation in this paper. Therefore, considering that there is no absolute clear boundary between similarity and dissimilarity of epidemics, this paper regards it as a fuzzy phenomenon, and therefore chooses a fuzzy mathematical model for prediction research. The concept of posting progress in fuzzy mathematics dealing with pattern recognition problems enables the determination of the weight of each reference epidemic; this weight is the degree of similarity between the selected five epidemic events and the COVID-19 epidemic. In the fuzzy mathematical model, the factors affecting the level of economic recovery are divided into two types: influencing factors and performance factors. The former mainly determine the size of the posting progress of the control epidemic and the target epidemic, and the latter are used to determine the degree of economic recovery of the control epidemic. The influencing factors include the basic information of the country and the information of the severity of the epidemic. The basic information comprises the size of the geographical area, population, and GDP, whilst the information of the severity of the epidemic comprises the scope, duration and the number of deaths. The performance factors are macroeconomic indicators and include non-pharmaceutical public health interventions such as government quarantine duration, consumer information. The influencing factors mainly include country-related information and information on the severity of the epidemic for the five major public health emergencies.

This paper gathers data from a total of 12 major public health emergencies that have occurred over the past 100 years in order to determine the five major public health emergencies. These included 1918 Spanish flu, 1953 Philippine dengue virus, 1974 Indian smallpox, 1997 British mad cow disease, 2001 British foot-and-mouth disease, 2003 Chinese SARS virus, 2007 Indonesian avian influenza (bird flu), 2009 Mexican swine flu, 2009 USA H1N1 flu, 2012 Middle East respiratory syndrome (MERS), 2014 West African Ebola virus, and 2016 Brazilian Zika virus. Whilst the Spanish flu pandemic was very similar to the COVID-19 epidemic in terms of impact and degree of harm, it was not selected because the relevant data needed for empirical evidence were not available. The Philippine dengue virus and the Indian smallpox virus epidemic were also abandoned because of the difficulty in obtaining data, and the British mad cow disease and the British foot-and-mouth disease were not selected because their degree of impact was too small. Existing literature infers that when using fuzzy mathematical models for economic forecasting of epidemics, it is better to have an epidemic in the fuzzy set that occurs in the same place as the forecast group, as this will improve the accuracy of the model. Therefore, the SARS epidemic in China was chosen. The Indonesian bird flu, Mexican swine flu, and USA H1N1 flu are all influenza epidemics. So the influential US H1N1 avian influenza outbreak was chosen to avoid duplication. The MERS, West African Ebola virus, and Brazilian Zika virus are more in line with the characteristics of the control epidemics. Therefore, our final five epidemics were the 2003 SARS virus in China, 2009 USA H1N1 flu, 2012 MERS, 2014 West African Ebola virus, and 2016 Brazilian Zika virus. They are relatively scattered in terms of the time and area of distribution, as expected from the selection.

Performance factors This paper identifies eight major macroeconomic indicators: CPI (Consumer Price Index); Resident Final Consumption; Government Final Consumption; Money Supply; Employed Population; Fiscal Revenue; Import Volume; Export Volume^26^. In order to avoid the influence of the inter-indicator scale, the data are selected before and after the epidemic, and use the rate of change to calculate. These indicators can both visualize the impact of the epidemic and indirectly reflect the impact of other factors or non-pharmaceutical health interventions^27,28^. For example, CPI can observe both the epidemic’s impact on the price changes of actual household expenditures and on the prices of goods and services throughout the economic recovery; household final consumption can observe household direct expenditures on goods and services and examine whether the epidemic has brought about anxiety and changes in people’s consumption behaviors, causing residents to reduce their consumption expenditures, which in turn may The economic recovery can also be measured by looking at the government's spending on public services, the central bank’s adjustments to the money, and the money supply data. Employment changes after the epidemic are also worthy of consideration as they can reflect the impact of the epidemic on the labor force, and thus its impact on economic recovery. The change in tax revenue can show the contribution of government support for SMEs after the epidemic and tax exemptions to economic recovery. Finally, the amount of imports and exports, as mentioned above, is proportional to the impact of the epidemic.

The data for each of the above indicators are obtained from the WIEGO Statistical Database, the World Health Organization, the BvD-EIU CountryData Database and the website of the National Bureau of Statistics of China.

### Modeling principle

Since it is not possible to accurately describe the similarities between the five past outbreaks and the COVID-19 outbreak, the concept of closeness in fuzzy mathematics is chosen to represent the similar distance between the control outbreak and the target outbreak. The exponential smoothing method for time series^16^ is then used to construct the weighting factors to approximate the recovery prediction of the Chinese economy after the occurrence of the COVID-19 epidemic in 2020.The model method of this article mainly refers to Huang's^6^ paper in 2012.

In the fuzzy mathematical model, *m* influencing factors (i.e., 8 macroeconomic indicators) that have a significant effect on economic recovery are selected. Using these *m* factors as criteria, *n* historical epidemics that are similar to the COVID-19 epidemic (i.e., five epidemic events) are selected as controls for prediction analysis. We applies the fuzzy set theory of fuzzy mathematics to select control epidemic weights. Whereby ${x}_{ij}$ denotes the value of the affiliation function of the $j$th characteristic’s influencing factor of the $i$th epidemic, where ${x}_{ij}\in [{0,1}]$^29^. The influence of the $n$th reference epidemic can be expressed as the following fuzzy characteristic matrix.

$$\left( {\begin{array}{*{20}l} {x_{{11}} } \hfill & \cdots \hfill & {x_{{1m}} } \hfill \\ \vdots \hfill & \ddots \hfill & \vdots \hfill \\ {x_{{n1}} } \hfill & \cdots \hfill & {x_{{nm}} } \hfill \\ \end{array} } \right) = \left( {X_{1} ,X_{2} , \ldots ,X_{n} } \right),x_{{ij}} \in [0,1]$$

Let us assume that ${x}_{j}^{*}$ is the value of the affiliation function of the th characteristic influence factor of the COVID-19 epidemic, and the characteristic fuzzy vector of the COVID-19 epidemic is

$${X}^{*}:\text{The} {X}^{*}=({x}_{1}^{*},{x}_{2}^{*},\ldots ,{x}_{m}^{*}),{x}_{j}^{*}\in [{0,1}]$$

The European closeness is chosen in this paper and calculated as follows:

1 $${\beta }_{i}=1-\frac{1}{\sqrt{n}}{\left[\sum_{j=1}^{m}{({x}_{ij}-{x}_{j}^{*})}^{2} \right]}^\frac{1}{2}, i={1,2},\ldots ,n$$

whereby ${\beta }_{i}$ denotes the closeness of the $i$th control outbreak to the COVID-19 epidemic, ${x}_{ij}$ and ${x}_{j}^{*}$ denote the affiliation function values of the control epidemic and the factors influencing the characteristics of the COVID-19 epidemic, respectively.We sort the closeness of the *n* control epidemics in ascending order, i.e., $({\beta }_{1},{\beta }_{2},\ldots ,{\beta }_{n})\to ({\beta }_{1}^{*},{\beta }_{2}^{*},\ldots ,{\beta }_{n}^{*})$, and then use exponential smoothing to forecast the post-epidemic economic recovery in China as follows:

2 $$\begin{aligned} S^{*} & = \beta _{n}^{*} s_{n} + \left( {1 - \beta _{n}^{*} } \right)s_{n}^{*} \\ & = \beta _{n}^{*} s_{n} + \left( {1 - \beta _{n}^{*} } \right)\left[ {\left( {\beta _{{n - 1}}^{*} s_{{n - 1}} + \left( {1 - \beta _{{n - 1}}^{*} } \right)s_{{n - 1}}^{*} } \right)} \right] \\ & = \cdots \\ & = \beta _{n}^{*} s_{n} + \left( {1 - \beta _{n}^{*} } \right)\beta _{{n - 1}}^{*} s_{{n - 1}} + ... + \left( {1 - \beta _{n}^{*} } \right)(1 - \beta _{{n - 1}}^{*} ) \cdots (1 - \beta _{2}^{*} )\beta _{1}^{*} s_{1} \\ \end{aligned}$$

whereby ${S}^{*}$ denotes the predicted economic integrated recovery for the COVID-19 epidemic, and ${s}_{i}$ denotes the integrated economic recovery for the 1-year period of the *i*th epidemic, and ${s}_{i}^{*}$ denotes the predicted integrated economic recovery for the 1-year period of the *i*th outbreak, satisfying ${s}_{j}^{*}={\beta }_{j-1}^{*}{s}_{j-1}+(1-{\beta }_{j-1}^{*}){s}_{j-1}^{*}(j={1,2},\ldots ,n)$ and take ${\beta }_{i}(i={1,2},\ldots ,n)$ as the smoothing coefficient of the index of the $i$th control epidemic. The average of the economic integrated recovery of the $n$th control epidemic is chosen as the initial condition in the recursive exponential smoothing equation, i.e., ${s}_{1}^{*}=\frac{\sum_{i=1}^{n}{\beta }_{i}{s}_{i}}{\sum_{i=1}^{n}{\beta }_{i}}$.

Then we use principal component analysis method in multivariate statistics^30^ to assess the comprehensive economic recovery degree. Firstly, the original matrix is constructed, and the $n$ epidemics similar to the current epidemic are taken as statistical samples and sorted in ascending order according to the magnitude of the closeness obtained earlier, whilst $p$ indicators are selected as performance factors in the 1-year period economic recovery examination to obtain the following raw data information Matrix.

$$\left( {\begin{array}{*{20}l} {t_{{11}} } \hfill & \cdots \hfill & {t_{{1p}} } \hfill \\ \vdots \hfill & \ddots \hfill & \vdots \hfill \\ {t_{{n1}} } \hfill & \cdots \hfill & {t_{{np}} } \hfill \\ \end{array} } \right) = (T_{1} ,T_{2} , \ldots ,T_{p} )$$

whereby ${t}_{ij}$ denotes the size of the $j$th performance factor indicator for the $i$th outbreak,${T}_{i}=({t}_{1i},{t}_{2i},...,{t}_{ni}{)}^{T},i={1,2},\ldots ,p$. We then calculate the covariance matrix of the control epidemic.$\sum ={({s}_{ij})}_{p\times p}$, i.e., ${s}_{ij}=\frac{1}{n-1}\sum_{k=1}^{n}({t}_{ki}-\overline{{t }_{i}})({t}_{kj}-\overline{{t }_{j}}),i,j={1,2},\ldots ,p$ Then find the eigenvalues of the covariance matrix: ${\lambda }_{1}\ge {\lambda }_{2}\ge \cdots \ge {\lambda }_{p}\ge 0$, and the corresponding orthogonalized unit eigenvectors:${a}_{i}=({a}_{1i},{a}_{2i},\ldots ,{a}_{ni}{)}^{T},i={1,2},\ldots ,p$. And the original data are standardized, i.e.

3 $${t}_{ij}^{*}=\frac{{t}_{ij}-\overline{{t }_{j}}}{{s}_{j}}(i={1,2},\ldots ,n;j={1,2},\ldots ,p)$$

whereby ${t}_{ij}^{*}$ denotes the $j$th performance factor of the $i$th control epidemic after dimensional standardization treatment. The standardized matrix is denoted as ${T}^{*}=\left({T}_{1}^{*},{T}_{2}^{*},\ldots ,{T}_{P}^{*}\right).$ The matrix is denoted as $\overline{{t }_{j}}$ denotes the sample mean of the $j$th performance factor of the control epidemics, i.e.$\overline{{t }_{j}}=\frac{1}{n}\sum_{i=1}^{n}{t}_{ij}$. The sample standard deviation of the $j$th performance factor in the *n* control epidemics is ${s}_{j}=\sqrt{\frac{1}{n-1}\sum_{i=1}^{n}({t}_{ij}-\overline{{t }_{j}}{)}^{2}}$. Based on the orthogonalized unit eigenvectors obtained in the second step ${a}_{i}$ and the normalization matrix ${T}^{*}$, the *m* principal component scores of the combined economic recovery of the *n* control epidemics can be calculated, i.e., using the variance contribution ratio ${a}_{i}=\frac{{\lambda }_{i}}{\sum_{k=1}^{p}{\lambda }_{k}}$ to explain the magnitude of the information reflected by the principal components, and *m* is determined by the cumulative contribution rate G(m) = $\sum_{i=1}^{m}{a}_{i}$ reached 90% or more as the principle. The calculation equation is as follows:

$${F}_{i}={a}_{1i}{T}_{1}^{*}+{a}_{2i}{T}_{2}^{*}+\cdots +{a}_{pi}{T}_{p}^{*},i={1,2},\ldots ,m$$

Finally, we use the variance contribution ratio as the weight and calculate the principal components ${F}_{1},{F}_{2},\ldots ,{F}_{m}$ of the weighted average as the control epidemic integrated economic recovery degree as follows.

4 $$S = \left[ {\begin{array}{*{20}l} {s_{1} } \hfill \\ {s_{2} } \hfill \\ \cdots \hfill \\ {s_{n} } \hfill \\ \end{array} } \right] = \left( {F_{1} ,F_{2} , \ldots ,F_{n} } \right)\left[ {\begin{array}{*{20}l} {a_{1}^{*} } \hfill \\ {a_{2}^{*} } \hfill \\ \cdots \hfill \\ {a_{n}^{*} } \hfill \\ \end{array} } \right]$$

whereby *S* denotes the control epidemic integrated economic recovery vector that satisfies $S=({s}_{1},{s}_{2},\ldots ,{s}_{n}{)}^{T}.{ a}_{i}^{*}$ denotes the weighted adjusted orthogonal unit eigenvector that satisfies ${a}_{i}^{*}=\frac{{a}_{i}}{\sum_{k=1}^{m}{a}_{k}},i={1,2},\ldots ,m$.

## Empirical analysis

Five epidemics that are similar to the COVID-19 epidemic were selected as the control epidemics to calculate the nearness degree of the control epidemics, amongst which only the more serious epidemics of Guinea, Sierra Leone, and Liberia were counted in the West African Ebola epidemic. Our selected five epidemics, three are respiratory infectious diseases (SARS, H1N1, MERS) that are similar to the COVID-19 epidemic, whilst the Ebola virus and Zika virus in Brazil, although not respiratory infectious diseases, can be compared with the COVID-19 epidemic in terms of their scope and intensity (see Appendix Table A1 for details). Based on the influencing factors in the hypothetical conditions, a statistical analysis was conducted for each control epidemic separately, and data were obtained by normalizing six characteristic factors amongst the countries, and the intensity of the impact of the epidemic at that time, namely, land area, population, GDP, epidemic impact, number of deaths, and epidemic duration (see Table A2 in the “Appendix” for details). Meanwhile, using the information collected from COVID-19 epidemic in China, the characteristic fuzzy vector of the COVID-19 epidemic under the six characteristic factors was obtained, notably ${X}^{*}$ = (1, 1, 0.482, 1, 0.328, 0.103). Using Eq. (1), the magnitude of the closeness of the five control epidemics can be derived and sorted in ascending order to obtain the following results.

5 $$\begin{aligned} (\beta _{1} ,\beta _{2} , \ldots ,\beta _{n} ) & = (0.5553,0.3544,0.174,0.337,0.44246) \\ & \quad \to (\beta _{1}^{*} ,\beta _{2}^{*} , \ldots ,\beta _{n}^{*} ) = (0.174,0.337,0.3544,0.44246,0.5553) \\ \end{aligned}$$

Specifically, the similarity between the 2003 SARS epidemic in China and the COVID-19 epidemic is 0.56, which is the highest out of the five control epidemic. The closeness between the 2009 H1N1 epidemic and the COVID-19 epidemic is 0.35, the closeness between the 2012 MERS and the COVID-19 epidemic is 0.17, and the closeness between the 2014 Ebola epidemic and the COVID-19 epidemic is 0.17. The closeness between the 2014 Ebola epidemic and the COVID-19 epidemic is 0.34. Finally, the closeness between the 2016 Zika epidemic and COVID-19 epidemic is 0.44. The second highest degree of similarity is with the 2016 Zika epidemic that infected 1.5 million people worldwide, and the breadth of transmission is very similar to that of the COVID-19 epidemic. However, the Zika epidemic was mainly in Latin America and other regions, while this COVID-19 epidemic has a much wider transmission range. The next selected epidemic was the H1N1 epidemic, which affected the USA and Mexico to a greater extent and was successfully controlled within a certain time frame, and so did not have the same impact as the COVID-19 epidemic. Whilst a few other countries were infected by the MERS and Ebola virus epidemics, it was the Middle East and West Africa that were hardest hit, and so the scope of infection was not as great as that of the COVID-19 epidemic and it did not cause as much economic damage, hence their closeness was relatively small.

Next, to calculate the comprehensive economic recovery against the epidemic in the semi-annual period, data on nine performance factor indicators were collected through the information for the semi-annual period after the five epidemics, and to avoid the influence of time factor and magnitude, the data were treated here for year-on-year rate of change, as shown in Table 1.

**Table 1:** Indicators of economic recovery performance factors in the six-month period after the control epidemic.

Control epidemic	CPI	Final consumption of the population	Employment	Fiscal revenue	Imports	Exports	Government final consumption	Money supply
Saudi Arabia (${X}_{1}^{*}$)	9.9	19.58	15.44	53.82	11.35	5.15	12.97	47.05
West Africa (${X}_{2}^{*}$)	16.46	3.1	5.49	18.71	1.82	36.54	11.11	36.69
United States (${X}_{3}^{*}$)	1.9	0.27	3.01	0.31	4.29	8.79	2.8	8.91
Brazil (${X}_{4}^{*}$)	7.89	− 2.42	2.48	2.82	4.09	7.54	− 0.18	3.43
China (${X}_{5}^{*}$)	3.06	24.79	6.33	40.83	43.63	46.33	45.19	25.49

Source: WIEGO statistical database and BvD-EIU CountryData database.

The R software is next used to perform a principal component analysis on the performance factors of the five epidemics (the results are detailed in Appendix Table A3). Appendix Table A3 indicates that the cumulative contribution of the first three principal components exceeds 98%, which can already represent the vast majority of information. The principal component expression (4) is then used to find the top three principal component scores for the five control epidemics (see Appendix Table A4). Finally, we predicted the recovery degree of this epidemic. Based on Eq. (4), we calculated the combined economic recovery degree of the five control epidemics as follows:

6 $${S}^{T}=({s}_{1},{s}_{2},\ldots ,{s}_{5}{)}^{T}=(-\text{0.329,0.880,0.436,0.922},-1.910{)}^{T}$$

The previously closeness magnitude obtained from Eq. (5) for these five control epidemics, and the integrated economic recovery obtained from Eq. (6) are brought together in Eq. (2) below to obtain the integrated economic recovery for COVID-19 epidemic.

$${S}^{*}={\beta }_{5}^{*}{s}_{5}+\left(1-{\beta }_{5}^{*}\right){\beta }_{4}^{*}{s}_{4}+ \cdots +\left(1-{\beta }_{5}^{*}\right)(1-{\beta }_{4}^{*})\cdots (1-{\beta }_{2}^{*}){{\beta }_{1}^{*}s}_{1}=-0.7994$$

In order to be able to describe the specific degree of recovery of this epidemic more accurately, 7 level degree indicators of recovery with reference to the level indicators in the analytic hierarchy process^5,31^. The level is shown in Table 2.

**Table 2:** Comprehensive recovery degree.

Scale	Meaning
I (− 2, − 1.25)	The degree of economic recovery is highly problematic and the economy tends to be in a prolonged slump
II [− 1.25, − 0.75)	The degree of economic recovery is not satisfactory, and it may take more than 1 year for the economy to gradually recover
III [− 0.75, − 0.25)	The degree of economic recovery is slow, but is expected to return to normal within 1 year
IV [− 0.25, 0.25)	The economy has largely recovered to its original level, but post-support work is still needed
V [0.25, 0.75)	The economy is not only back to normal, but is on a normal economic development track
VI [0.75, 1.25)	The economic recovery exceeds expectations, and there is steady post-disaster economic growth
VII [1.25, 2)	Rapid economic growth due to disasters

**Figure 1:**
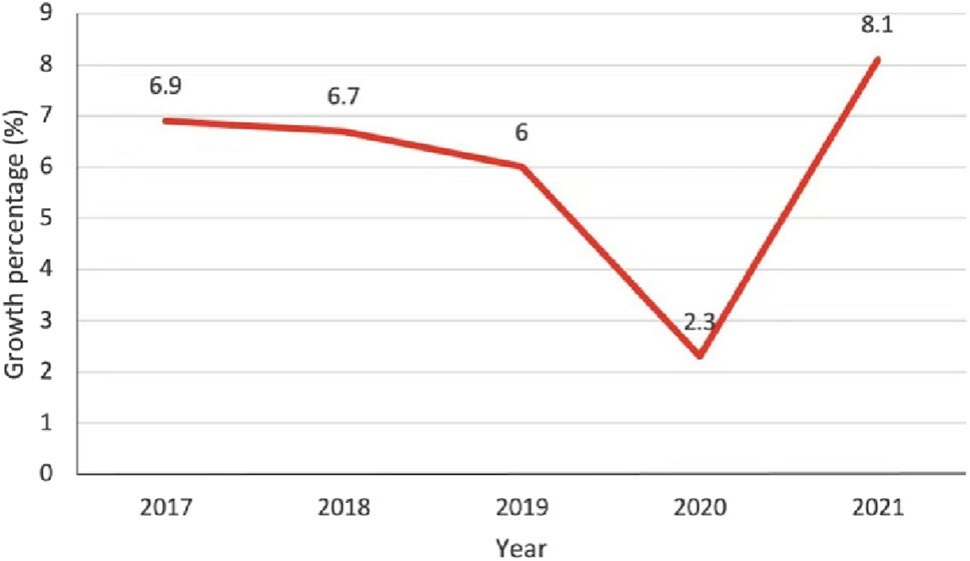
China’s GDP Growth Rate (2017–2021). Source: WIEGO statistical database.

According to Table 2, it can be judged that the combined impact of the COVID-19 epidemic on the Chinese economy should be on the second scale, but the numerical results are very close to the third scale, indicating that the predicted degree of recovery for China’s economy is not particularly positive, and the economy may take more than 1 year to return to normal. This result is actually still basically in line with the reality of China's economy after the end of the epidemic. As the epidemic improved, the Communist Party Central Committee introduced a series of economic stimulus measures, including the distribution of a large number of consumption vouchers to the public and the lifting of travel restrictions before the May 1 2020 Golden Week. Local governments have also been introducing local stimulus packages and supporting small, medium, and micro enterprises. Through active efforts in April and May 2020 and the so-called “retaliatory consumption” of the population in the post-epidemic era, China’s economy has made a V-shaped reversal (see Fig. 1), where it is clear that the GDP growth rate has dropped from 6% in 2019 to 2.3% in 2020, which is sufficient to indicate the serious impact of the epidemic on the Chinese economy. With the strong stimulus and help from the Chinese government, the GDP growth rate reached 8.1% in 2021, which was basically back to the pre-epidemic level.

This paper now forecasts individual economic indicators through an economic forecasting model. The results are shown in Table 3. Residential final consumption belongs to the fourth scale, that is, it has recovered to the original degree, but still needs follow-up policy support and stimulation. Due to various ways of stimulating consumption by the government, which has indeed driven a large amount of consumption. However, this paper indicates that government intervention has seen a steady improvement in residential consumption, but that it has not reached the level of basic recovery. CPI belongs to the second scale. The impact of the employed population is also considerable, and the forecast belongs to the first scale, i.e., the decline of the employed population will be a long-term phenomenon, and the epidemic has indeed greatly impacted the job market, with people in many industries losing their jobs and graduates having difficulties in finding jobs, which is more in line with reality; fiscal revenue, currency The fiscal revenue, money supply and export volume all belong to the second scale, i.e., the degree of recovery is not very satisfactory, and it takes at least 1 year to recover, and it is difficult for China’s export volume to recover in the short term due to the current impact of the international epidemic, which also warns us that we should shift our economic development to domestic and not rely too much on exports. The results of this forecast tell us that the COVID-19 epidemic is ongoing and that foreign countries are affected by the epidemic and also need to export a large amount of goods to reduce the losses from the epidemic, and that the recovery of these two components will soon return to pre-epidemic levels.

**Table 3:** Forecast of individual economic performance factors.

Economic scale	Recovery	Scale
CPI (consumer price index)	− 0.89	Second scale [II]
Resident final consumption	− 0.05	Fourth scale [IV]
Employed population	− 1.42	First scale [I]
Financial revenue	− 1.17	Second scale [II]
Import amount	− 0.63	Third scale [III]
Export amount	− 0.89	Second scale [II]
Government final consumption	− 0.6	Third scale [III]
Money supply	− 0.94	Second scale [II]

Overall, the model prediction results show that although China suffered a very serious economic shock throughout the COVID-19 epidemic, active state intervention and scientific and effective economic stimulus, have enabled the economy to recover to pre-epidemic levels within 1 year, which is in line with the actual situation, and validates the prediction model.

## Conclusion

This paper uses fuzzy mathematics to select five historical major public health emergencies similar to the COVID-19 epidemic and predicts China’s economic recovery as well as eight individual economic indicators for the COVID-19 epidemic. The model predicts that composite economic indicators (CEI) will reach the pre-epidemic level in about 1 year relative to 2020, and the predicted results are consistent with the actual situation. Amongst the individual economic indicators, final consumption of the population is the fastest to recover, followed by final government consumption and imports, followed by CPI, fiscal revenue, exports and money supply; the slowest recovery is within the employment sector. The predicted results are all in line with China’s 2020 economic and social development, indicating the validity of this prediction model. In light of the abovementioned conclusions this paper offers the following policy suggestions.

The COVID-19 epidemic remains a global “public health emergency” according to the World Health Organization (WHO), and continues to create a complex international social environment, and necessitates an increase in global trade protection measures from China’s government. A change is required in the economic development pattern, with particular focus on developing the domestic market. based on the domestic and international "double cycle" development strategy, make up for the integrity of the supply chain and industrial chain in the national market, but also actively participate in the global industrial chain and develop the international market, adhere to the “One Belt, One Road” strategy and cooperation. At the same time, it should be noted that the post-epidemic era of physical mode globalization has changed to digital and smart globalization. Therefore, China should continue to develop digital technology, digital economy and digital industry, and continuously develop e-commerce and the Internet industry. Equally, in the face of trade protection, China should pursue the path of independent innovation. This requires the government to continue to give concessions to science and technology innovation enterprises, in order to ultimately achieve high-quality economic and social development.

The Chinese government should introduce a series of strong policies to accelerate the recovery of MSMEs through measures such as fee reduction, and debt deferment. The tertiary sector has been most affected by the epidemic, with wholesale and retail trade, accommodation and catering experiencing the most severe losses with 13–35% and 32–40% year-on-year decline in value added, respectively. Equally, the government must introduce measures to effectively prevent and control the epidemic and realize the smooth development of industrial chains, labor service chains and supply chains, while giving MSMEs a series of phased policies on fee reduction and social security fee reduction, as well as policies to defer debt and solve financing difficulties.

The job market has suffered great losses during the epidemic and so the development of jobs is crucial to both stabilizing people’s livelihoods and preserving employment, in turn aiding the economic recovery and securing long-term development. This can be achieved in part through making full use of the advantages of e-commerce and "Internet+" to develop online employment. The epidemic has also caused huge losses to farmers in the distribution of agricultural products and greenhouses, and migrant workers are also unable to find jobs due to the economic downturn in the city. The government should increase subsidies and insurance to help farmers overcome these difficulties.

The development of China’s economy cannot be separated from consumption, and promoting consumption is the favorable magic weapon for economic growth. For bulk commodities, consumption tax can be adopted, and for the retail industry, coupons and promotions can be issued to stimulate consumption. In addition to introducing government measures at all levels, the recovery of consumer confidence is also key. Following the epidemic, consumers will have palpitations about the consumption of food and beverage, air transportation and tourism, so making consumers feel at ease and improving consumer confidence is also the key to economic recovery.

This paper does also have some limitations. For example a more comprehensive study of the factors affecting economic recovery could be considered. The authors will continue to search for the missing data related to the 1918 Spanish flu, and will update the paper if any new findings are made.

## Supplementary Information

Supplementary Information.

## Data Availability

The datasets used and analysed during the current study available from the corresponding author on reasonable request.
