# Supplementary material for: Analysis of economic forecasting in the post-epidemic era: evidence from China
Source: Sci Rep. 2023 Feb 15;13:2696. doi: 10.1038/s41598-022-19011-z (PMC9930051; doi:10.1038/s41598-022-19011-z)
Supplement: Supplementary file 1 — Supplementary Information. [file 41598_2022_19011_MOESM1_ESM.docx]

**Appendix.**

**Table A1 Control epidemic samples**

| Country | Epidemic | Time |
| --- | --- | --- |
| China | SARS Epidemic (SARS) | 2003 |
| United States | Influenza outbreak (H1N1) | 2009 |
| Saudi Arabia | Middle East Respiratory Syndrome (MERS) | 2012 |
| West Africa | Ebola virus (EBOLA) | 2014 |
| Brazil | Zika virus (ZIKA) | 2016 |

Source: World Health Organization.

**Table A2 Table of affiliation function values for the control epidemic**

| **Characteristic factors** | **Country area** | **Population** | **GDP** | **Impact range** | **Number of deaths** | **Duration** |
| --- | --- | --- | --- | --- | --- | --- |
| China ($X_{1}$) | 1 | 0.917 | 0.115 | 0.135 | 0.05 | 0.205 |
| United States ($X_{2}$) | 0.972 | 0.219 | 1 | 0.2 | 1 | 0.436 |
| Saudi Arabia ($X_{3}$) | 0.234 | 0.021 | 0.052 | 0.121 | 0.032 | 1 |
| West Africa ($X_{4}$) | 0.662 | 0.158 | 0．001 | 0.047 | 0.8 | 0.256 |
| Brazil ($X_{5}$) | 0.887 | 0.146 | 0.125 | 0.2 | 0.133 | 0.205 |

Source: WIEGO statistical database, World Health Organization.

**Table A3 Table of variance contribution of principal components**

| **Principal component** | **Principal component standard deviation** | **Variance contribution** | **Cumulative contribution** |
| --- | --- | --- | --- |
| 1 | 2 | 0.5 | 0.5 |
| 2 | 1.67 | 0.35 | 0.855 |
| 3 | 1.04 | 0.13 | 0.989 |
| 4 | 0.29 | 0.01 |  |

**Table A4 Table of principal component scores**

| control group  epidemic | First principal component | Second principal component | Third principal component |
| --- | --- | --- | --- |
| Saudi Arabia ($X_{1}^{*}$ ) | 1.17 | -2.21 | -1.08 |
| West Africa ($X_{2}^{*}$ ) | 1.65 | -0.47 | 1.58 |
| United States ($X_{3}^{*}$ ) | -0.04 | 1.57 | -0.71 |
| Brazil ($X_{4}^{*}$ ) | 0.64 | 1.78 | -0.16 |
| China ($X_{5}^{*}$ ) | -3.42 | -0.67 | 0.37 |
